# Supplementary material for: Feasibility, acceptability, and safety of a novel device for self-collecting capillary blood samples in clinical trials in the context of the pandemic and beyond
Source: PLoS One. 2024 May 29;19(5):e0304155. doi: 10.1371/journal.pone.0304155 (PMC11135758; doi:10.1371/journal.pone.0304155)
Supplement: S1 Table — Reprinted from [25] under a CC BY license, with permission from Tasso Inc., original copyright [2020]. (DOCX) [file pone.0304155.s001.docx]

| **Training Appreciation:** | **Responses** |
| --- | --- |
| After watching the instructional video, how comfortable are you with the prospect of using the TASSO device on yourself? | 1- Not at all comfortable 2- Slightly comfortable 3- Moderately comfortable 4- Very comfortable 5- Extremely comfortable |
| After watching the instructional video, how useful was the brochure? | 1- Not at all useful  2- Slightly useful 3- Moderately useful 4- Very useful 5- Extremely useful |
| Do you feel ready to use the device on yourself? | 1- Yes, I feel ready 0-No, I don’t feel ready |
| Which instructional materials are important for us to provide for at-home blood sampling? | 1- Video only 2- Brochure only 3- Both video and brochure |
| For labelling and preparing the samples for shipment, how easy to understand were the instructions from the video and the written instructions | 1- Very difficult 2- Difficult 3- Neutral 4- Easy 5- Very easy |
| How useful was the guidance of the research assistant when you used the device for the first time? | 1- Not at all useful 2- Slightly useful 3- Moderately useful 4- Very useful 5- Extremely useful |
| **Acceptability:** | |
| Overall, how would you rate the experience of using this new device for blood extraction? | 1- Very negative 2- Negative 3- Neutral 4- Positive 5- Very positive |
| If you had the choice to visit the hospital for blood collection, how likely would you select home blood sampling over having blood collected using a needle at the hospital? | 1- Extremely unlikely 2- Unlikely 3- No preference 4- Likely 5- Extremely likely |

## **Table S1. Questionnaire ascertaining training appreciation and acceptability in the adult study of the Investigational Phase**
