# Supplementary material for: Feasibility, acceptability, and safety of a novel device for self-collecting capillary blood samples in clinical trials in the context of the pandemic and beyond
Source: PLoS One. 2024 May 29;19(5):e0304155. doi: 10.1371/journal.pone.0304155 (PMC11135758; doi:10.1371/journal.pone.0304155)
Supplement: S2 Table — (DOCX) [file pone.0304155.s002.docx]

## **Table S2. Questionnaire ascertaining training appreciation and acceptability in the child-parent dyad study of the Investigational Phase**

| **Question** | **Responses** |
| --- | --- |
| **Training Appreciation:** | |
| (Child) How useful was the video on how to use a TASSO-SST device on a child? | 1- Not at all useful 2- Slightly useful 3- Moderately useful 4- Very useful 5- Extremely useful |
| (Child) After watching the instructional video, how useful was the brochure? | 1- Not at all useful 2- Slightly useful 3- Moderately useful 4- Very useful 5- Extremely useful |
| (Parent) After having watched the video and read the brochure, do you feel ready to use the device on yourself? | 1- Yes 0- No |
| (Parent) Which distraction method will you be using to keep your child calm and distracted? ? | 1-Sugar water 2- Letting your child play the most entertaining game on your phone/tablet  3- Listening to pleasant music  4- Singing a song  5- Telling a happy story/having the child tell a story  6- Discussing the child's favourite activities  7- Watching a TV show  8- Other: ____________________ 9- None |
| (Child) How useful was the distraction (mention which one)? | 1- Not at all useful 2- Slightly useful 3- Moderately useful 4- Very useful 5- Extremely useful |
| (Parent) Was the method of distraction you chose easily applicable during the blood draw? | 1- Yes 0- No |
| (Parent) Now that you have already used the device once on your child, should a research assistant be available for guidance if you were to use it again? | 1- Yes 0- No |
| **Acceptability:** | |
| (Child) Would you prefer to use the TASSO-SST device first or would you prefer to watch your parent/sibling use the TASSO-SST device first? | 1- I would prefer to use the TASSO-SST device first  2- I would prefer to watch my parent/sibling use the TASSO-SST device first |
| (Parent) Would you prefer to test the device on yourself first? | 1- Yes 0- No |
| (Parent) If the TASSO device on your child collected an insufficient volume of blood on your first try, would you have accepted to use the device on your child a second time? | 1- Yes 0- No |
| (Parent) Overall, how acceptable was the sampling procedure using the TASSO for your child? | 1- Totally unacceptable 2- Unacceptable 3- Neutral 4- Acceptable 5- Totally acceptable |
| (Parent) How likely would you select a home blood sampling over having a blood sampling using a needle at the hospital for your child? | 1- Extremely unlikely 2- Unlikely 3- No preference 4- Likely 5- Extremely likely |
