# Supplementary material for: Feasibility, acceptability, and safety of a novel device for self-collecting capillary blood samples in clinical trials in the context of the pandemic and beyond
Source: PLoS One. 2024 May 29;19(5):e0304155. doi: 10.1371/journal.pone.0304155 (PMC11135758; doi:10.1371/journal.pone.0304155)
Supplement: S3 Table — (DOCX) [file pone.0304155.s003.docx]

## **Table S3. Patient-reported outcomes**

|  | **Investigational - Adult** | | **Investigational - Child-Parent Dyad** | | **Implementation** | |
| --- | --- | --- | --- | --- | --- | --- |
|  |  | | **Children^*^** | **Parents^†^** | **PROTECT** | **DIVA** |
|  | N=90 | | N=9 | N=9 | N= 15 adults | N= 2 Children |
| **Training Appreciation** |  | |  |  |  |  |
| **Instructional Brochure, n (%)** |  | |  |  |  |  |
| *After watching the instructional video, how useful was the brochure?* | Not at all useful - 4 (4.4) Slightly useful - 17 (18.9) Moderately useful - 25 (27.8) Very useful - 27 (30)  Extremely useful - 17 (18.9) | | Not at all useful - 2 (25) Slightly useful - 5 (62.5) Moderately useful - 1 (12.5) Very useful - 0 (0)  Extremely useful - 0 (10) | — | — | — |
| **Instructional Video, n (%)** |  |  |  |  |  |  |
| **Question** | *After watching the instructional video, how comfortable are you with the prospect of using the TASSO device on yourself?* | | *How useful was the video on how to use a TASSO-SST device on a child?* | *After watching the instructional video and reading the instructional brochure, how comfortable are you with the prospect of using the TASSO device on your child?* | — | — |
| Response | Not at all comfortable - 0 (0) Slightly comfortable - 1 (1.11) Moderately comfortable - 6 (6.7) Very comfortable - 48 (53.3)  Extremely comfortable - 25 (38.9) | | Not at all useful - 5 (62.5) Slightly useful - 2 (25) Moderately useful - 1 (12.5) Very useful - 0 (0)  Extremely useful - 0 (10) | Not at all comfortable - 4 (44.4) Slightly comfortable - 4 (44.4) Moderately comfortable - 1 (11.1) Very comfortable - 0 (0)  Extremely comfortable – 0 (0) | — | — |
| **Preferred instructional format, n (%)** |  | |  |  |  |  |
| Video | 11 (12.22) | | — | — | — | — |
| Brochure | 7 (7.78) | | — | — | — | — |
| Both video and brochure | 72 (80) | | — | — | — | — |
| **Clarity of shipping instructions, n (%)** |  | |  |  |  |  |
| *For labelling and preparing the samples for shipment, how easy to understand were the instructions from the video and the written instructions?* | Very difficult - 0 (0) Difficult - 0 (0) Neutral - 2 (2.3) Easy - 32 (36)  Very easy - 55 (61.8) | | — | — | — | — |
| **Usefulness of live guidance, n (%)** |  | |  |  |  |  |
| Question | *How useful was the guidance of the research assistant when you used the device for the first time?* | | — | *Now that you have already used the device on your child, should a research assistant be available for guidance if you were to use it again? (Yes or No)* | — | — |
| Response | Not at all useful - 4 (4.5) Slightly useful - 11 (12.4) Somewhat useful - 20 (22.5) Moderately useful – 12 (13.5)  Extremely useful - 42 (47.2) | | — | Yes- 6 (66.6) No- 3(33.3) | — | — |
| **Distraction usefulness, n (%)^‡^** |  | |  |  |  |  |
| Question | — | | *How useful was the distraction ?* | *Was the method of distraction you chose easily applicable during the blood draw?* | — | — |
| Response | — | | Not at all useful - 0 (0) Slightly useful - 0 (0) Moderately useful - 2 (25) Very useful - 0 (0)  Extremely useful - 0 (10) | Yes- 9(100) | — | — |
| **Readiness to use the device, n (%)*** |  | |  |  |  |  |
| *Do you feel ready to use the device?* | Yes- 90 (100) | | Yes- 9 (100) | Yes- 7 (100) | — | — |
| **Acceptability** |  | |  |  |  |  |
| **Overall Satisfaction** |  | |  |  |  |  |
| Question | *Overall, how would you rate the experience of using this new device for blood extraction?* | | — | *Overall, how acceptable was the sampling procedure using the TASSO for your child?* | — | — |
| Response | Very negative - 0 (0) Negative - 1 (1.1) Neutral - 2 (2.3) Positive - 22 (24.7)  Very positive - 64 (71.9) | | — | Totally unacceptable - 1 (11.1) unacceptable - 0 (0) Neutral - 0 (0) Acceptable - 0 (0)  Perfectly acceptable- 9 (88.8) | — | — |
| **Willingness to use again, n(%)** |  | |  |  |  |  |
| *Would you use the device again? (Yes or No)* | Yes- 90 (100) | | — | Yes- 7 (100) | Yes - 15 (100) | Yes - 2 (100) |
| **Preference over venipuncture, n (%)** |  | |  |  |  |  |
| *If you had the choice to visit the hospital for blood collection, how likely would you select home blood sampling over having blood collected using a needle at the hospital ?* | Extremely unlikely - 0 (0) Unlikely - 3 (3.4) No preference - 5 (5.6) Likely - 22 (24.7)  Extremely likely - 59 (66.3) | | — | Extremely unlikely - 2 (22.2) Unlikely - 0 (0) No preference - 1 (11.1) Likely - 1 (11.1)  Extremely likely - 5 (55.6) | — | — |
| * Although 9 children participated in the child-parent dyad study; responses from children > 4 years of age were collected. † Although 9 parents as part of 9 child-parent dyad participated in the study, some questions pertaining to blood collection in children were answered by all 9 parents; however only 7 out of 9 parents chose to try the device for capillary blood collection, so questions pertaining to blood collection on themselves were reported as n (%) among these. ‡ To collect blood from children, a distraction method was employed; children aged 4 and above (8 out of 9) were asked to respond, while parents of children under 4 years were asked about the applicability of the chosen distraction method. | | | | | | |
