# Supplementary material for: Feasibility, acceptability, and safety of a novel device for self-collecting capillary blood samples in clinical trials in the context of the pandemic and beyond
Source: PLoS One. 2024 May 29;19(5):e0304155. doi: 10.1371/journal.pone.0304155 (PMC11135758; doi:10.1371/journal.pone.0304155)
Supplement: S1 Fig — (PDF) [file pone.0304155.s004.pdf]

FAQ (Frequently Asked Questions)

How does the TASSO device work?

The TASSO Button sticks to the skin with a light adhesive. When the button is pressed, vacuum forms and a small lancet pricks the surface of the skin. The vacuum draws blood out of the capillaries and into a sample pod attached to the bottom of the TASSO Button.

What is the best position on my arm to get a good sample?

The best placement for your device is on the outside of your upper arm, three finger-widths below the top of your shoulder.

I pressed the button, but I don't see blood.

If it's only been a minute or two, please relax. It takes a while for the blood to start flowing.

I pressed the button more than two minutes ago and I still don't see blood

First, let's make sure you pressed the button all the way. Press it again firmly. If you get a big click and you feel a small prick, then start the five-minute timer again and wait for your blood sample.

If you don't get a big click when you press it again, then either your arm was not warm enough, the device wasn't placed quite right, or there is a problem with the device. Please contact us our research team and we will send you a new kit.

FAQ (continued)

How many times can each TASSO device be used?

Each device is used only once to collect a sample and then should be discarded.

How many times can someone sample in a single day using different TASSO devices?

There is no set limit to the number of samples someone can collect in a single day. In studies, TASSO devices have been used up to 8 times on the same patient in the same day.

Does it hurt to collect blood with the TASSO device?

Participants in clinical studies have reported lower levels of pain with the TASSO device compared to collecting blood with a finger stick or standard venipuncture procedure.

What now?

Proceed with the blood test and send it to us.

Please take some time to fill out the questionnaire to let us know about your experience.

Other questions?

Please feel free to contact your research coordinator.

How to use the TASSO device

Instructions, images and devices kindly provided by Tasso, Inc.

Pamphlet prepared for the

ValiCap2020 study (Validation of capillary home blood sampling)

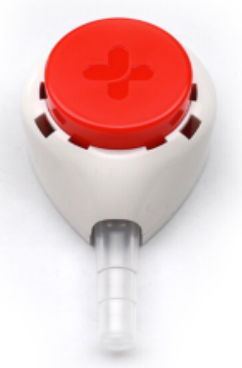

Thank you for your participation!

Centre de recherche CHU Sainte-Justine
Le centre hospitalier universitaire mère-enfant

Université de Montréal

Version 1.0, 07 08 2020

Hello! This is the TASSO device.

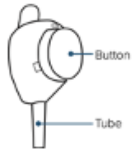

For a video of instructions, please go to:

www.tassoinc.com/tasso-sst-video

Preparation

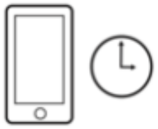

1. Get a timer.

You'll need it for step 9.

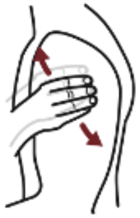

2. Rub arm quickly and firmly just below the shoulder.

Rub vigorously for about 30 seconds until it's very warm to help your blood flow.

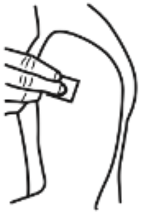

3. Clean arm with alcohol pad.

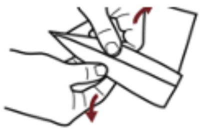

4. Open device pouch by pulling apart white and clear layers like a bag of potato chips.

Collection

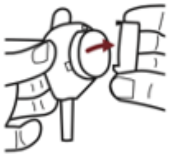

5. Remove clear plastic cover over the red button.

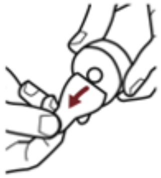

6. Peel paper tab behind the red button.

Keep tube pointing down.

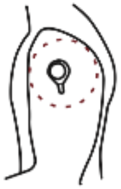

7. Stick device to shoulder.

Do not remove once it's on.

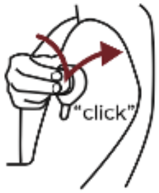

8. Press button quickly and firmly until it can't go any farther.

Wait 2 seconds before letting the button go.

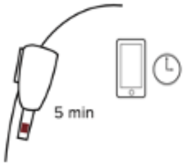

9. Start a five-minute timer.

Keep your arm at your side. You won't see blood right away.

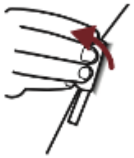

10. After 5 minutes (or earlier if tube fills), peel off TASSO device.

No need to fill the tube completely.

Return to Research Team

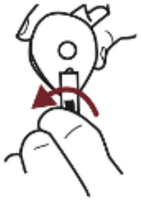

11. Remove tube by firmly twisting a quarter turn and pulling down.

Please grip it tightly and twist 1/4 turn.

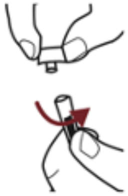

12. Twist cap fully onto the tube.

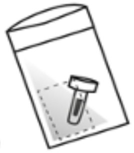

13. Place tube in the clear Biohazard bag, seal, and stick the research label on the bag.

Leave the absorbent pad in the bag.

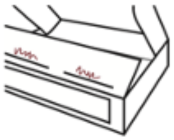

14. Write collection date and time on the inside flap of the box (day-month-year hour:minutes).

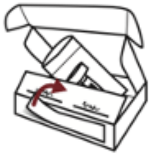

15. Place bag in box and seal with the strip.

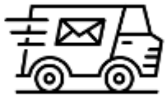

16. Ship box within 24 hours of packing.

Throw out used TASSO button into standard household trash.
