# Supplementary material for: Feasibility, acceptability, and safety of a novel device for self-collecting capillary blood samples in clinical trials in the context of the pandemic and beyond
Source: PLoS One. 2024 May 29;19(5):e0304155. doi: 10.1371/journal.pone.0304155 (PMC11135758; doi:10.1371/journal.pone.0304155)
Supplement: S2 Fig — (PDF) [file pone.0304155.s005.pdf]

S2 Figure. Reported pain by puncture site and topical analgesia use in the Adult Investigational Phase.

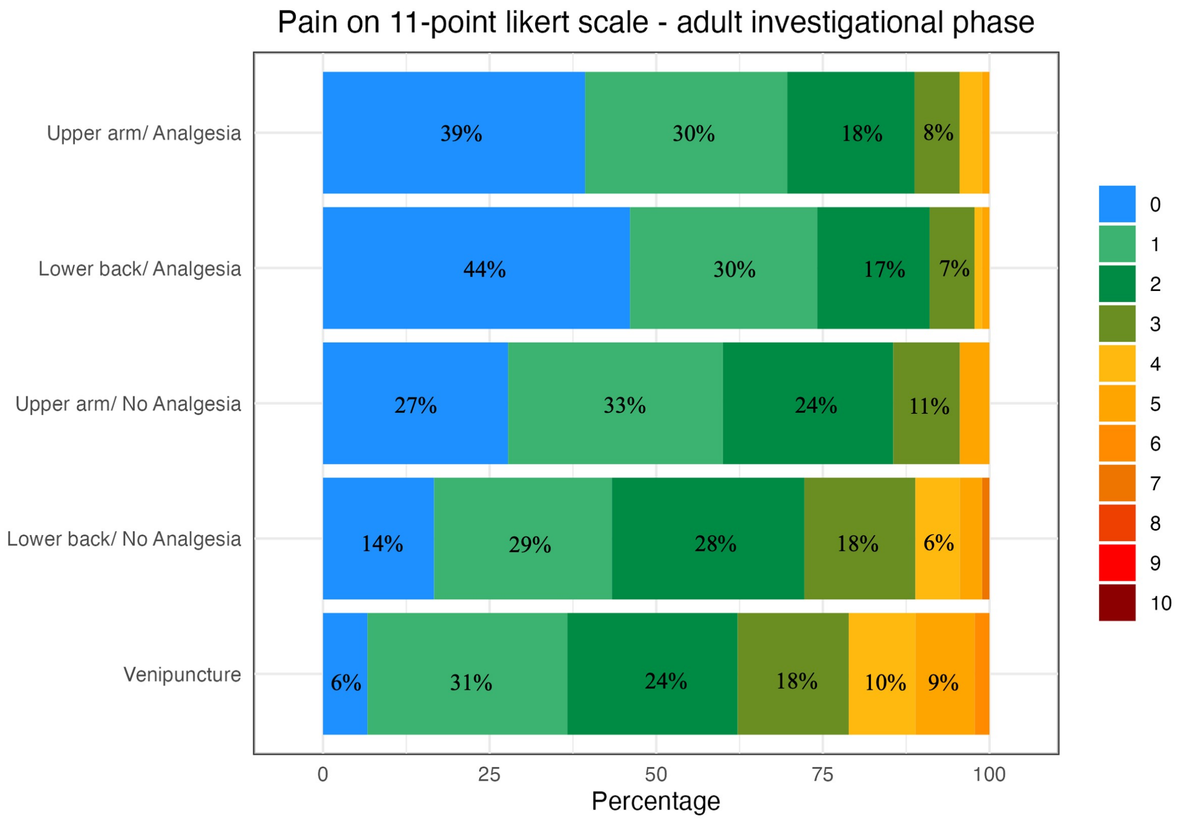

Participants in the study used the Louisiana Pain Scale to describe their levels of pain.(1)
Pain is reported on the 11-point Likert scale from 0 (blue): no pain to 10 (chestnut): worst pain.

Detailed description of the Louisiana Pain Scale is as follows:

0- You don't feel any pain at all.
1- You barely notice this kind of pain, and sometimes you don't even think of it.
2- Your pain is minor but can get annoying at times.
3- Your pain is somewhat distracting, but you can get used to it and function.
4- This level is considered moderate pain; this means it is distracting, and only if you are deeply involved in an activity, you might be able to ignore it for a little bit.
5- You won't be able to ignore this moderately strong pain for longer than a few minutes; working or participating in social activities will take some effort on your part.
6- You will have difficulties concentrating with this moderately strong pain; this type of pain will interfere with your daily activities and routines.

7- You will be very limited when it comes to performing your daily activities. This moderate to severe pain will dominate your senses. You will have a hard time sleeping.
8- When experiencing this intense pain, you won't be able to move much, if at all. You will also have a hard time engaging in conversations with others.
9- This excruciating will make you cry and moan uncontrollably.
10- This unspeakable pain will make you bedridden and possibly even delirious.

1. Specialists LP. Pain Scale : help your doctors understand you [Available from: https://www.louisianapain.com/blog/pain-scale-help-your-doctor-understand-you.
