# Supplementary material for: Feasibility, acceptability, and safety of a novel device for self-collecting capillary blood samples in clinical trials in the context of the pandemic and beyond
Source: PLoS One. 2024 May 29;19(5):e0304155. doi: 10.1371/journal.pone.0304155 (PMC11135758; doi:10.1371/journal.pone.0304155)
Supplement: S3 Fig — (PDF) [file pone.0304155.s006.pdf]

Figure S3. Distribution of crude pain on the 11-point Louisiana pain scale among children and accompanying parents in the child-parent dyad study

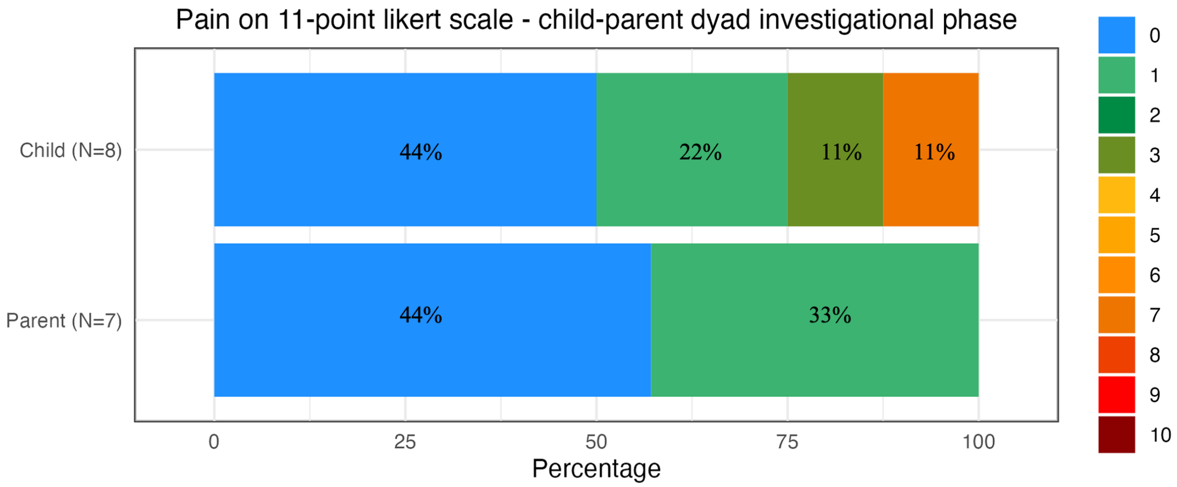

Participants in the study used the Louisiana Pain Scale to describe their levels of pain.(1)

Out of the 9 children recruited, 8 reported their pain scores. The remaining participant, who was under 4 years old, had the pain ascertained by his/her parent and was not included in the graph. Of the 9 parents recruited for the child-parent dyad investigational phase, 7 of them used the TASSO-SST device on themselves.

1. Specialists LP. Pain Scale : help your doctors understand you [Available from: https://www.louisianapain.com/blog/pain-scale-help-your-doctor-understand-you.
