# Supplementary material for: Feasibility, acceptability, and safety of a novel device for self-collecting capillary blood samples in clinical trials in the context of the pandemic and beyond
Source: PLoS One. 2024 May 29;19(5):e0304155. doi: 10.1371/journal.pone.0304155 (PMC11135758; doi:10.1371/journal.pone.0304155)
Supplement: Attachment — Submitted filename: point by point response _PONE 2024-02-07 (1).dotx [file pone.0304155.s007.dotx]

**Feasibility, acceptability, and safety of a novel device for self-collecting capillary blood samples in clinical trials in the context of the pandemic and beyond [PONE-D-23-28982]**

1. Please upload a Response to Reviewers letter, which should include a point-by-point response to each of the points made by the Editor and/or Reviewers. (This should be uploaded as a 'Response to Reviewers' file type.) Please follow this link for more information: <http://blogs.PLOS.org/everyone/2011/05/10/how-to-submit-your-revised-manuscript/>

**A point-by-point response to the Editor and/or Reviewers was provided with our previous submission on December 20^th^, 2024, titled as “point by point response _PONE 2023-12-2023”. Point-by-point responses will again be uploaded as a file titled “Response to Reviewers” to avoid confusion.**

2. Please note that funding information should not appear in the Acknowledgments section/Funding section or any other areas of your Manuscript. We will only publish funding information present in the Funding Statement section of the online submission form. Please remove any funding-related text from the manuscript.

**We have removed the funding information in the manuscript, originally included before the acknowledgement section in the previous version. The clean version, now titled ”Implementation Manuscript_2024-02-07_Clean,” will be uploaded as a clean version, and the tracked changes version titled “Implementation Manuscript_2024-02-07_with tracked changes” will be uploaded as part of the current submission.**

3. In this instance it seems there may be acceptable restrictions in place that prevent the public sharing of your minimal data.PLOS requires that the minimal data for your study be made available upon request in this case. However, in line with our goal of ensuring long-term data availability to all interested researchers, PLOS' Data Policy states that authors cannot be the sole named individuals responsible for ensuring data access (<http://journals.plos.org/plosone/s/data-availability#loc-acceptable-data-sharing-methods>).

Data requests to a non-author institutional point of contact, such as a data access or ethics committee, help guarantee long-term stability and availability of data. Providing interested researchers with a durable point of contact ensures data will be accessible even if an author changes email addresses or institutions or becomes unavailable to answer requests.

Before we proceed with your manuscript, please address the following prompts in an updated and complete Data Availability statement:

If there are ethical or legal restrictions on sharing a de-identified data set, please explain the restrictions in detail (e.g., data containing potentially identifying or sensitive patient information) and who has imposed them (e.g., a Research Ethics Committee or Institutional Review Board, etc.). Please also provide non-author contact information (phone/email/hyperlink) for a data access committee, ethics committee, or other institutional body to which data requests may be sent. If applicable, please also provide any necessary information that interested researchers would need when requesting access to data to obtain the minimal data set for your study.

We will update your Data Availability statement on your behalf to reflect the information you provide.

**Thank you for your clarification. We have updated our Data availability statement as below:**

**The dissemination of data to the public is precluded due to an omission pertaining to the inclusion of a data-sharing provision within the consent forms, as mandated by the Research Ethics Board. Anonymized Data are available from the CHU Sainte Justine Institutional Data Access / Ethics Committee (contact via ethique.hsj@ssss.gouv.qc.ca) for researchers who meet the criteria for access to confidential data.**
